# Supplementary material for: The Cardiometabolic Health of African Immigrants in High-Income Countries: A Systematic Review
Source: Int J Environ Res Public Health. 2022 Jun 29;19(13):7959. doi: 10.3390/ijerph19137959 (PMC9265760; doi:10.3390/ijerph19137959)
Supplement: Supplementary file 1 [file ijerph-19-07959-s001.zip › ijerph-1773136-supplementary.pdf]

Supplementary Materials

The Cardiometabolic Health of African Immigrants in High-Income Countries: A Systematic Review

Danielle Mensah[1#], Oluwabunmi Ogungbe[2#], Ruth-Alma N. Turkson-Ocran[3], Chioma Onuoha[4], Samuel Byiringiro[2], Nwakaego A. Nmezi[5], Ivy Mannoh[6], Elisheva Wecker[6], Ednah N Madu[7], Yvonne Commodore-Mensah[2,8*]
Correspondence: Yvonne Commodore-Mensah, ycommod1@jhmi.edu

Table S1: PRISMA Checklist

Section and Topic	Item #	Checklist item	Location where item is reported
**TITLE**			
Title	1	Identify the report as a systematic review.	1
**ABSTRACT**			
Abstract	2	See the PRISMA 2020 for Abstracts checklist.	1
**INTRODUCTION**			
Rationale	3	Describe the rationale for the review in the context of existing knowledge.	2
Objectives	4	Provide an explicit statement of the objective(s) or question(s) the review addresses.	2
**METHODS**			
Eligibility criteria	5	Specify the inclusion and exclusion criteria for the review and how studies were grouped for the syntheses.	3
Information sources	6	Specify all databases, registers, websites, organisations, reference lists and other sources searched or consulted to identify studies. Specify the date when each source was last searched or consulted.	2
Search strategy	7	Present the full search strategies for all databases, registers and websites, including any filters and limits used.	2
Selection process	8	Specify the methods used to decide whether a study met the inclusion criteria of the review, including how many reviewers screened each record and each report retrieved, whether they worked independently, and if applicable, details of automation tools used in the process.	2-4
Data collection process	9	Specify the methods used to collect data from reports, including how many reviewers collected data from each report, whether they worked independently, any processes for obtaining or confirming data from study investigators, and if applicable, details of automation tools used in the process.	2-4
Data items	10a	List and define all outcomes for which data were sought. Specify whether all results that were compatible with each outcome domain in each study were sought (e.g. for all measures, time points, analyses), and if not, the methods used to decide which results to collect.	2-4
	10b	List and define all other variables for which data were sought (e.g., participant and intervention characteristics, funding sources). Describe any assumptions made about any missing or unclear information.	NA
Study risk of bias assessment	11	Specify the methods used to assess risk of bias in the included studies, including details of the tool(s) used, how many reviewers assessed each study and whether they worked independently, and if applicable, details of automation tools used in the process.	3, Figure S1
Effect measures	12	Specify for each outcome the effect measure(s) (e.g., risk ratio, mean difference) used in the synthesis or presentation of results.	3-4
Synthesis methods	13a	Describe the processes used to decide which studies were eligible for each synthesis (e.g., tabulating the study intervention characteristics and comparing against the planned groups for each synthesis (item #5)).	4
	13b	Describe any methods required to prepare the data for presentation or synthesis, such as handling of missing summary statistics, or data conversions.	3-4
	13c	Describe any methods used to tabulate or visually display results of individual studies and syntheses.	2-6

Section and Topic	Item #	Checklist item	Location where item is reported
	13d	Describe any methods used to synthesize results and provide a rationale for the choice(s). If meta-analysis was performed, describe the model(s), method(s) to identify the presence and extent of statistical heterogeneity, and software package(s) used.	NA
	13e	Describe any methods used to explore possible causes of heterogeneity among study results (e.g., subgroup analysis, meta-regression).	NA
	13f	Describe any sensitivity analyses conducted to assess robustness of the synthesized results.	NA
Reporting bias assessment	14	Describe any methods used to assess risk of bias due to missing results in a synthesis (arising from reporting biases).	3, Figure S1
Certainty assessment	15	Describe any methods used to assess certainty (or confidence) in the body of evidence for an outcome.	3, Figure S1
**RESULTS**			
Study selection	16a	Describe the results of the search and selection process, from the number of records identified in the search to the number of studies included in the review, ideally using a flow diagram.	4-5 Figure 1
	16b	Cite studies that might appear to meet the inclusion criteria, but which were excluded, and explain why they were excluded.	Figure 1
Study characteristics	17	Cite each included study and present its characteristics.	Table 1
Risk of bias in studies	18	Present assessments of risk of bias for each included study.	3, Figure S1
Results of individual studies	19	For all outcomes, present, for each study: (a) summary statistics for each group (where appropriate) and (b) an effect estimate and its precision (e.g. confidence/credible interval), ideally using structured tables or plots.	Table S2
Results of syntheses	20a	For each synthesis, briefly summarise the characteristics and risk of bias among contributing studies.	Figure S1
	20b	Present results of all statistical syntheses conducted. If meta-analysis was done, present for each the summary estimate and its precision (e.g. confidence/credible interval) and measures of statistical heterogeneity. If comparing groups, describe the direction of the effect.	8-12
	20c	Present results of all investigations of possible causes of heterogeneity among study results.	NA
	20d	Present results of all sensitivity analyses conducted to assess the robustness of the synthesized results.	NA
Reporting biases	21	Present assessments of risk of bias due to missing results (arising from reporting biases) for each synthesis assessed.	Figure S1
Certainty of evidence	22	Present assessments of certainty (or confidence) in the body of evidence for each outcome assessed.	Figure S1
**DISCUSSION**			
Discussion	23a	Provide a general interpretation of the results in the context of other evidence.	13-14
	23b	Discuss any limitations of the evidence included in the review.	13-14
	23c	Discuss any limitations of the review processes used.	13-14
	23d	Discuss implications of the results for practice, policy, and future research.	13-14
**OTHER INFORMATION**			
Registration and protocol	24a	Provide registration information for the review, including register name and registration number, or state that the review was not registered.	2
	24b	Indicate where the review protocol can be accessed, or state that a protocol was not prepared.	2
	24c	Describe and explain any amendments to information provided at registration or in the protocol.	2
Support	25	Describe sources of financial or non-financial support for the review, and the role of the funders or sponsors in the review.	15
Competing interests	26	Declare any competing interests of review authors.	15
Availability of data, code and other materials	27	Report which of the following are publicly available and where they can be found: template data collection forms; data extracted from included studies; data used for all analyses; analytic code; any other materials used in the review.	2

Table S2: Database Search Strategy

Topic	Cardiometabolic Health of African Immigrants in High-Income Countries
Databases	PubMed, Embase.com, CINAHL Plus, Cochrane Library via Wiley, Scopus, Web of Science
Reference Management	EndNote, Covidence
Date Run	July 19, 2021
Total Results	17,951
Duplicates Removed	9,293
Remaining Results	8,658

PubMed<br>3,217 results July 19, 2021
("Emigrants and Immigrants"[Mesh] OR "Emigration and Immigration"[Mesh] OR "Transients and Migrants"[Mesh] OR "African born"[tw] OR alien[tw] OR aliens[tw] OR "asylum seek*"[tw] OR "birth place"[tw] OR birthplace[tw] OR "border cross*"[tw] OR "cross border*"[tw] OR diaspora*[tw] OR emigrant[tw] OR emigrants[tw] OR emigrat*[tw] OR "foreign-born*"[tw] OR "foreign national*"[tw] OR "foreign worker*"[tw] OR foreigner*[tw] OR illegals[tw] OR immigrant[tw] OR immigrants[tw] OR immigrat*[tw] OR migrant*[tw] OR migrat*[tw] OR nomad[tw] OR nomads[tw] OR refugee[tw] OR refugees[tw] OR squatter[tw] OR squatters[tw] OR undocumented[tw])<br>AND<br>("Africa"[Mesh] OR Africa*[tw] OR Algeria*[tw] OR Angola*[tw] OR Basutoland*[tw] OR Bechuanaland*[tw] OR Benin*[tw] OR black[tw] OR blacks[tw] OR Botswana*[tw] OR "Burkina Faso*"[tw] OR "Burkina Fasso*"[tw] OR Burundi*[tw] OR "Cabo Verde*"[tw] OR Cameroon*[tw] OR Cameroun*[tw] OR "Cape Verde*"[tw] OR Centrafrica*[tw] OR Centrafriqu*[tw] OR Chad*[tw] OR "Comoro Island*"[tw] OR Comoros*[tw] OR Congo*[tw] OR "Cote D'Ivoire*"[tw] OR "Cote Dilvoire*"[tw] OR Dahome*[tw] OR Djibouti*[tw] OR Egypt*[tw] OR Eritrea*[tw] OR Ethiopia*[tw] OR Eswatini*[tw] OR Gabon*[tw] OR Gaboon*[tw] OR Gambia*[tw] OR Ghana*[tw] OR "Gold Coast*"[tw] OR Guinea*[tw] OR Ifni*[tw] OR libia*[tw] OR "Issa land"[tw] OR "Ivory Coast*"[tw] OR Kalahari*[tw] OR Katanga*[tw] OR Kenya*[tw] OR Lesothan*[tw] OR Lesotho*[tw] OR Liberia*[tw] OR Libya*[tw] OR Madagasc*[tw] OR Maghreb*[tw] OR Maghrib*[tw] OR "Malagasy Republic"[tw] OR Malawi*[tw] OR Mali[tw] OR Malian*[tw] OR Mauretania*[tw] OR Mauritania*[tw] OR Mayott*[tw] OR Mocambiqu*[tw] OR Morocc*[tw] OR Mozambiqu*[tw] OR Namibia*[tw] OR negro[tw] OR negroes[tw] OR negros[tw] OR "NHB"[tw] OR "NHBs"[tw] OR Niger*[tw] OR Nigeria*[tw] OR Nyasaland*[tw] OR "of color"[tw] OR "of colour"[tw] OR Rhodesia*[tw] OR "Rio Muni*"[tw] OR Ruanda*[tw] OR Rwanda*[tw] OR Sahara*[tw] OR Sahel*[tw] OR "Sao Tome and Principe*"[tw] OR Senegal*[tw] OR "Sierra Leone*"[tw] OR Somali*[tw] OR Sudan*[tw] OR Swaziland*[tw] OR Tanganyika*[tw] OR Tanzania*[tw] OR Tchad*[tw] OR Togo*[tw] OR Tunesia*[tw] OR Tunisia*[tw] OR "Ubangi Shari*"[tw] OR Uganda*[tw] OR "United Arab Republic*"[tw] OR "Upper Volta*"[tw] OR Urundi*[tw] OR Zaire*[tw] OR Zambia*[tw] OR Zanzibar*[tw] OR Zimbabwe*[tw])<br>AND<br>("Cardiometabolic Risk Factors"[Mesh] OR "Cardiovascular Diseases"[Mesh] OR "Cardiovascular Infections"[Mesh] OR "Diabetes Mellitus"[Mesh] OR "Dyslipidemias"[Mesh] OR "Fused Kidney"[Mesh] OR "Glucose Metabolism Disorders"[Mesh] OR "Glycogen Storage Disease"[Mesh] OR "Heart"[Mesh] OR "Heart Disease Risk Factors"[Mesh] OR "Insulin Resistance"[Mesh] OR "Kidney"[Mesh] OR "Kidneys, Artificial"[Mesh] OR "Kidney Diseases"[Mesh] OR "Kidney Neoplasms"[Mesh] OR "Kidney Transplantation"[Mesh] OR "Metabolic Syndrome"[Mesh] OR "National Institute of Diabetes and Digestive and Kidney Diseases (U.S.)"[Mesh] OR "Nephrostomy, Percutaneous"[Mesh] OR "Nicotine"[Mesh] OR "Overweight"[Mesh] OR "Renal Replacement Therapy"[Mesh] OR "Smokers"[Mesh] OR "Smoking"[Mesh] OR "Smoking Cessation"[Mesh] OR "Smoking Cessation Agents" [Pharmacological Action] OR "Smoking Devices"[Mesh] OR "Smoking Prevention"[Mesh] OR "Smoking Reduction"[Mesh] OR "Tobacco"[Mesh] OR "Tobacco Use Cessation Devices"[Mesh] OR "abnormal blood pressure"[tw] OR "abnormal blood pressures"[tw] OR "adipose tissue hyperplas*"[tw] OR adiposity[tw] OR aneurysm[tw] OR aneurysms[tw] OR antihypertensi*[tw] OR anuria[tw] OR aorta[tw] OR aortic[tw] OR aortitis[tw] OR aortopulmonary[tw] OR arrhythmia[tw] OR arrhythmias[tw] OR arterial[tw] OR arteriosclerosis[tw] OR arteriosus[tw] OR arteriovenous[tw] OR arteritis[tw] OR asystol*[tw] OR atherogenesis[tw] OR atheromatosis[tw] OR atheroscler*[tw] OR atria[tw] OR atrial[tw] OR atrioventricular[tw] OR atrium[tw] OR bicuspid[tw] OR "blood dialyzer"[tw] OR "blood dialyzers"[tw] OR "blood pressure anomaly"[tw] OR "blood pressure anomalies"[tw] OR BMI[tw] OR BMIs[tw] OR "body mass index*"[tw] OR "body weight"[tw] OR bradycardia*[tw] OR bradycardic[tw] OR cardiac[tw] OR cardio*[tw] OR carditis[tw] OR cigar[tw] OR cigars[tw] OR cigarette[tw] OR cigarettes[tw] OR cigarillo[tw] OR cigarillos[tw] OR coronary[tw] OR corpulence[tw] OR corpulency[tw] OR corpulent[tw] OR CRRT[tw] OR CRRTs[tw] OR CVD[tw] OR CVDs[tw] OR "decreased blood pressure"[tw] OR "Dent's disease"[tw] OR "depressed blood pressure"[tw] OR dextrocardia*[tw] OR diabet*[tw] OR diafiltration[tw] OR diafiltrations[tw] OR dialyses[tw] OR dialysis[tw] OR dialyzer[tw] OR dialyzers[tw] OR diastol*[tw] OR "disorder of carbohydrate metabolism"[tw] OR "disorders of carbohydrate metabolism"[tw] OR "disorder of glycosylation"[tw] OR "disorders of glycosylation"[tw] OR dyslipaemia*[tw] OR dyslipemia[tw] OR dyslipemias[tw] OR dyslipidaemia*[tw] OR dyslipidemia*[tw] OR dyslipoidosis[tw] OR dyslipoproteinemia*[tw] OR "dysmetabolic"[tw] OR "e-cig"[tw] OR "e-cigs"[tw] OR "ecig"[tw] OR "ecigs"[tw] OR eclampsia[tw] OR "elevated blood pressure"[tw] OR "elevated blood pressures"[tw] OR "elevated cholesterol*"[tw] OR "elevated triglyceride"[tw] OR "elevated triglycerides"[tw] OR embolism[tw] OR embolisms[tw] OR endarteritis[tw] OR endocard*[tw] OR endomyocard*[tw] OR epicard*[tw] OR "fat overload syndrome"[tw] OR "fructose intolerance"[tw] OR "glomerular necros*"[tw] OR glomerulitis[tw] OR glomeruloneph*[tw] OR glomerulopath*[tw] OR glomeruloscleroses[tw] OR glomerulosclerosis[tw] OR glomerulus[tw] OR "glucose intolerance"[tw] OR "glucose tolerance impairment"[tw] OR "glycogen storage disease"[tw] OR "glycogen storage diseases"[tw] OR glycogenoses[tw] OR glycogenosis[tw] OR "glycosylation disorder"[tw] OR "glycosylation disorders"[tw] OR glycosuria*[tw] OR glycosuric[tw] OR haemodiafiltration[tw] OR haemodiafiltrations[tw] OR haemodialysis[tw] OR haemofiltration[tw] OR haemofiltrations[tw] OR heart[tw] OR hearts[tw] OR hematuria*[tw] OR hemodiafiltration[tw] OR hemodiafiltrations[tw] OR hemodialyser[tw] OR hemodialysers[tw] OR hemodialyses[tw] OR hemodialysis[tw] OR hemodialyzer[tw] OR hemodialyzers[tw] OR hemofiltration[tw] OR hemofiltrations[tw] OR hemopericardium[tw] OR hepatorenal[tw] OR "high blood pressure"[tw] OR "high blood

pressures"[tw] OR "high cholesterol*"[tw] OR hydronephro*[tw] OR "hyper cholesteremia*"[tw] OR hypercholesteremia*[tw] OR "hyper cholesterol*"[tw] OR hypercholesterol*[tw] OR hyperemia*[tw] OR hyperglycem*[tw] OR "hyper glycem*"[tw] OR hyperinsulin*[tw] OR "hyper insulin*"[tw] OR hyperlipidemia[tw] OR hyperlipidemias[tw] OR hyperlipemia[tw] OR hyperlipemias[tw] OR hypernephroma*[tw] OR hypernephroid[tw] OR "hyper tension*"[tw] OR hypertension*[tw] OR hypertensive*[tw] OR "hyper triglyceridemia"[tw] OR hypertriglyceridemia[tw] OR "hyper triglyceridemias"[tw] OR hypertriglyceridemias[tw] OR hypertrophic[tw] OR hypertrophy[tw] OR "impaired glucose tolerance"[tw] OR "increased blood pressure"[tw] OR "insulin coma*"[tw] OR "insulin resistance"[tw] OR "insulin resistances"[tw] OR "insulin sensitivit*"[tw] OR "insulin shock*"[tw] OR interatrial[tw] OR interventricular[tw] OR intracardiac[tw] OR IRRT[tw] OR IRRTs[tw] OR kidney*[tw] OR lipedema*[tw] OR lipemia*[tw] OR lipidaemia*[tw] OR lipidema*[tw] OR lipidemia*[tw] OR lipoedema*[tw] OR "lipo proteinemia*"[tw] OR lipoproteinemia*[tw] OR "metabolic"[tw] OR "metabolism disorder"[tw] OR "metabolism disorders"[tw] OR moyamoya[tw] OR myocard*[tw] OR myopericard*[tw] OR nephrect*[tw] OR nephrit*[tw] OR nephro*[tw] OR nicoderm[tw] OR nicotine[tw] OR nicotinic[tw] OR obes*[tw] OR overweight*[tw] OR "over weight*"[tw] OR pacemaker[tw] OR pacemakers[tw] OR parasystole[tw] OR parasystolic[tw] OR periarterial[tw] OR periarteritis[tw] OR pericard*[tw] OR perineph*[tw] OR perirenal*[tw] OR PIRRT[tw] OR PIRRTs[tw] OR polyarteritis[tw] OR prediabet*[tw] OR prehypertens*[tw] OR "prosthetic valve"[tw] OR "prosthetic valves"[tw] OR pulmonary[tw] OR pyeloneph*[tw] OR "QT syndrome"[tw] OR quadricuspid[tw] OR renal*[tw] OR reninoma*[tw] OR renocardiac[tw] OR renodialysis[tw] OR "reno scleroses"[tw] OR renoscleroses[tw] OR "reno sclerosis"[tw] OR renosclerosis[tw] OR renotubular[tw] OR "reno tubular"[tw] OR renovascular[tw] OR "reno vascular"[tw] OR RRT[tw] OR RRTs[tw] OR sinoatrial*[tw] OR smoke[tw] OR smoker[tw] OR smokers[tw] OR smokes[tw] OR smoking[tw] OR snuff[tw] OR snuffing[tw] OR snuffs[tw] OR stroke[tw] OR strokes[tw] OR subaort*[tw] OR supraventric*[tw] OR "syndrome x"[tw] OR systol*[tw] OR tachycardia*[tw] OR tachycardic[tw] OR thromboembolism*[tw] OR tobacco*[tw] OR triatrium[tw] OR tricuspid[tw] OR thrombosis[tw] OR ultrafiltration*[tw] OR univentricular[tw] OR uremia*[tw] OR uremic[tw] OR urinoma*[tw] OR vape[tw] OR vaper[tw] OR vapers[tw] OR vapes[tw] OR vaping[tw] OR "vascular disease"[tw] OR "vascular diseases"[tw] OR "vascular disorder"[tw] OR "vascular disorders"[tw] OR "vascular necroses"[tw] OR "vascular necrosis"[tw] OR "vascular neoplasm"[tw] OR "vascular neoplasms"[tw] OR vasculitis[tw] OR vasculopath*[tw] OR ventricular[tw])

Embase
4,427 results July 19, 2021

('immigrant'/exp OR 'migrant'/exp OR 'migration'/exp OR 'african born':ti,ab,kw OR alien:ti,ab,kw OR aliens:ti,ab,kw OR 'asylum seek*':ti,ab,kw OR 'birth place':ti,ab,kw OR birthplace:ti,ab,kw OR 'border cross*':ti,ab,kw OR 'cross border*':ti,ab,kw OR diaspora*:ti,ab,kw OR emigrant:ti,ab,kw OR emigrants:ti,ab,kw OR emigrat*:ti,ab,kw OR 'foreign-born*':ti,ab,kw OR 'foreign national*':ti,ab,kw OR 'foreign worker*':ti,ab,kw OR foreigner*:ti,ab,kw OR illegals:ti,ab,kw OR immigrant:ti,ab,kw OR immigrants:ti,ab,kw OR immigrat*:ti,ab,kw OR migrant*:ti,ab,kw OR migrat*:ti,ab,kw OR nomad:ti,ab,kw OR nomads:ti,ab,kw OR refugee:ti,ab,kw OR refugees:ti,ab,kw OR squatter:ti,ab,kw OR squatters:ti,ab,kw OR undocumented:ti,ab,kw)
AND
('africa'/exp OR africa*:ti,ab,kw OR algeria*:ti,ab,kw OR angola*:ti,ab,kw OR basutoland*:ti,ab,kw OR bechuanaland*:ti,ab,kw OR benin*:ti,ab,kw OR black:ti,ab,kw OR blacks:ti,ab,kw OR botswana*:ti,ab,kw OR 'burkina faso*':ti,ab,kw OR 'burkina fasso*':ti,ab,kw OR burundi*:ti,ab,kw OR 'cabo verde*':ti,ab,kw OR cameroon*:ti,ab,kw OR cameroun*:ti,ab,kw OR 'cape verde*':ti,ab,kw OR centrafrica*:ti,ab,kw OR centrafriqu*:ti,ab,kw OR chad*:ti,ab,kw OR 'comoro island*':ti,ab,kw OR comoros*:ti,ab,kw OR congo*:ti,ab,kw OR 'cote dilvoire*':ti,ab,kw OR ((cote NEXT/3 ivoire*):ti,ab,kw) OR dahome*:ti,ab,kw OR djibouti*:ti,ab,kw OR egypt*:ti,ab,kw OR eritrea*:ti,ab,kw OR ethiopia*:ti,ab,kw OR eswatini*:ti,ab,kw OR gabon*:ti,ab,kw OR gaboon*:ti,ab,kw OR gambia*:ti,ab,kw OR ghana*:ti,ab,kw OR 'gold coast*':ti,ab,kw OR guinea*:ti,ab,kw OR ifni*:ti,ab,kw OR iibia*:ti,ab,kw OR 'issa land':ti,ab,kw OR 'ivory coast*':ti,ab,kw OR kalahari*:ti,ab,kw OR katanga*:ti,ab,kw OR kenya*:ti,ab,kw OR lesothan*:ti,ab,kw OR lesotho*:ti,ab,kw OR liberia*:ti,ab,kw OR libya*:ti,ab,kw OR madagasc*:ti,ab,kw OR maghreb*:ti,ab,kw OR maghrib*:ti,ab,kw OR 'malagasy republic':ti,ab,kw OR malawi*:ti,ab,kw OR mali:ti,ab,kw OR malian*:ti,ab,kw OR mauretania*:ti,ab,kw OR mauritania*:ti,ab,kw OR mayott*:ti,ab,kw OR mocambiqu*:ti,ab,kw OR morocc*:ti,ab,kw OR mozambiqu*:ti,ab,kw OR namibia*:ti,ab,kw OR negro:ti,ab,kw OR negroes:ti,ab,kw OR negros:ti,ab,kw OR 'nhb':ti,ab,kw OR 'nhbs':ti,ab,kw OR niger*:ti,ab,kw OR nigeria*:ti,ab,kw OR nyasaland*:ti,ab,kw OR 'of color':ti,ab,kw OR 'of colour':ti,ab,kw OR rhodesia*:ti,ab,kw OR 'rio muni*':ti,ab,kw OR ruanda*:ti,ab,kw OR rwanda*:ti,ab,kw OR sahara*:ti,ab,kw OR sahel*:ti,ab,kw OR 'sao tome and principe*':ti,ab,kw OR senegal*:ti,ab,kw OR 'sierra leone*':ti,ab,kw OR somali*:ti,ab,kw OR sudan*:ti,ab,kw OR swaziland*:ti,ab,kw OR tanganyika*:ti,ab,kw OR tanzania*:ti,ab,kw OR tchad*:ti,ab,kw OR togo*:ti,ab,kw OR tunesia*:ti,ab,kw OR tunisia*:ti,ab,kw OR 'ubangi shari*':ti,ab,kw OR uganda*:ti,ab,kw OR 'united arab republic*':ti,ab,kw OR 'upper volta*':ti,ab,kw OR urundi*:ti,ab,kw OR zaire*:ti,ab,kw OR zambia*:ti,ab,kw OR zanzibar*:ti,ab,kw OR zimbabwe*:ti,ab,kw)
AND
('cardiometabolic risk'/exp OR 'cardiovascular disease'/exp OR 'cardiovascular infection'/exp OR 'cardiovascular risk'/exp OR 'cardiovascular risk factor'/exp OR 'diabetes mellitus'/exp OR 'dialyzer'/exp OR 'disorders of carbohydrate metabolism'/exp OR 'dyslipidemia'/exp OR 'fused kidney'/exp OR 'glycogen storage disease'/exp OR 'heart'/exp OR 'heart disease risk factor'/exp OR 'insulin resistance'/exp OR 'kidney'/exp OR 'kidney disease'/exp OR 'kidney surgery'/exp OR 'kidney transplantation'/exp OR 'kidney tumor'/exp OR 'mesoblastic nephroma'/exp OR 'metabolic syndrome x'/exp OR 'nicotine'/exp OR 'obesity'/exp OR 'percutaneous nephrostomy'/exp OR 'renal replacement therapy'/exp OR 'smoking'/exp OR 'smoking cessation'/exp OR 'smoking cessation agent'/exp OR 'smoking device'/exp OR 'smoking prevention'/exp OR 'smoking reduction'/exp OR 'tobacco'/exp OR 'abnormal blood pressure':ti,ab,kw OR 'abnormal blood pressures':ti,ab,kw OR 'adipose tissue hyperplas*':ti,ab,kw OR adiposity:ti,ab,kw OR aneurysm:ti,ab,kw OR aneurysms:ti,ab,kw OR antihypertensi*:ti,ab,kw OR anuria:ti,ab,kw OR aorta:ti,ab,kw OR aortic:ti,ab,kw OR aortitis:ti,ab,kw OR aortopulmonary:ti,ab,kw OR arrhythmia:ti,ab,kw OR arrhythmias:ti,ab,kw OR arterial:ti,ab,kw OR arteriosclerosis:ti,ab,kw OR arteriosus:ti,ab,kw OR arteriovenous:ti,ab,kw OR arteritis:ti,ab,kw OR asystol*:ti,ab,kw OR atherogenesis:ti,ab,kw OR atheromatosis:ti,ab,kw OR atheroscler*:ti,ab,kw OR atria:ti,ab,kw OR atrial:ti,ab,kw OR atrioventricular:ti,ab,kw OR atrium:ti,ab,kw OR bicuspid:ti,ab,kw OR 'blood dialyzer':ti,ab,kw OR 'blood dialyzers':ti,ab,kw OR 'blood pressure anomaly':ti,ab,kw OR 'blood pressure anomalies':ti,ab,kw OR bmi:ti,ab,kw OR bmis:ti,ab,kw OR 'body mass index*':ti,ab,kw OR 'body weight':ti,ab,kw OR bradycardia*:ti,ab,kw OR bradycardic:ti,ab,kw OR cardiac:ti,ab,kw OR cardio*:ti,ab,kw OR carditis:ti,ab,kw OR cigar:ti,ab,kw OR cigars:ti,ab,kw OR cigarette:ti,ab,kw OR cigarettes:ti,ab,kw OR cigarillo:ti,ab,kw OR cigarillos:ti,ab,kw OR coronary:ti,ab,kw OR corpulence:ti,ab,kw OR corpulency:ti,ab,kw OR corpulent:ti,ab,kw OR crrt:ti,ab,kw OR crrts:ti,ab,kw OR cvd:ti,ab,kw OR cvds:ti,ab,kw OR 'decreased blood pressure':ti,ab,kw OR ((dent NEXT/3 disease):ti,ab,kw) OR 'depressed blood pressure':ti,ab,kw OR dextrocardia*:ti,ab,kw OR diabet*:ti,ab,kw OR diafiltration:ti,ab,kw OR diafiltrations:ti,ab,kw OR dialyses:ti,ab,kw OR dialysis:ti,ab,kw OR dialyzer:ti,ab,kw OR dialyzers:ti,ab,kw OR diastol*:ti,ab,kw OR 'disorder of carbohydrate metabolism':ti,ab,kw OR 'disorders of carbohydrate metabolism':ti,ab,kw OR 'disorder of glycosylation':ti,ab,kw OR 'disorders of glycosylation':ti,ab,kw OR dyslipaemia*:ti,ab,kw OR dyslipemia:ti,ab,kw OR dyslipemias:ti,ab,kw OR dyslipidaemia*:ti,ab,kw OR dyslipidemia*:ti,ab,kw OR dyslipoidosis:ti,ab,kw OR dyslipoproteinemia*:ti,ab,kw OR 'dysmetabolic':ti,ab,kw OR 'e-cig':ti,ab,kw OR 'e-cigs':ti,ab,kw OR 'ecig':ti,ab,kw OR 'ecigs':ti,ab,kw OR eclampsia:ti,ab,kw OR 'elevated blood pressure':ti,ab,kw OR 'elevated blood pressures':ti,ab,kw OR 'elevated cholesterol*':ti,ab,kw OR 'elevated triglyceride':ti,ab,kw OR 'elevated triglycerides':ti,ab,kw OR embolism:ti,ab,kw OR embolisms:ti,ab,kw OR endarteritis:ti,ab,kw OR endocard*:ti,ab,kw OR

endomyocard*:ti,ab,kw OR epicard*:ti,ab,kw OR 'fat overload syndrome':ti,ab,kw OR 'fructose intolerance':ti,ab,kw OR 'glomerular necros*':ti,ab,kw OR glomerulitis:ti,ab,kw OR glomeruloneph*:ti,ab,kw OR glomerulopath*:ti,ab,kw OR glomeruloscleroses:ti,ab,kw OR glomerulosclerosis:ti,ab,kw OR glomerulus:ti,ab,kw OR 'glucose intolerance':ti,ab,kw OR 'glucose tolerance impairment':ti,ab,kw OR 'glycogen storage disease':ti,ab,kw OR 'glycogen storage diseases':ti,ab,kw OR glycogenoses:ti,ab,kw OR glycogenosis:ti,ab,kw OR 'glycosylation disorder':ti,ab,kw OR 'glycosylation disorders':ti,ab,kw OR glycosuria*:ti,ab,kw OR glycosuric:ti,ab,kw OR haemodiafiltration:ti,ab,kw OR haemodiafiltrations:ti,ab,kw OR haemodialysis:ti,ab,kw OR haemofiltration:ti,ab,kw OR haemofiltrations:ti,ab,kw OR heart:ti,ab,kw OR hearts:ti,ab,kw OR hematuria*:ti,ab,kw OR hemodiafiltration:ti,ab,kw OR hemodiafiltrations:ti,ab,kw OR hemodialyser:ti,ab,kw OR hemodialysers:ti,ab,kw OR hemodialyses:ti,ab,kw OR hemodialysis:ti,ab,kw OR hemodialyzer:ti,ab,kw OR hemodialyzers:ti,ab,kw OR hemofiltration:ti,ab,kw OR hemofiltrations:ti,ab,kw OR hemopericardium:ti,ab,kw OR hepatorenal:ti,ab,kw OR 'high blood pressure':ti,ab,kw OR 'high blood pressures':ti,ab,kw OR 'high cholesterol*':ti,ab,kw OR hydronephro*:ti,ab,kw OR 'hyper cholesteremia*':ti,ab,kw OR hypercholesteremia*:ti,ab,kw OR 'hyper cholesterol*':ti,ab,kw OR hypercholesterol*:ti,ab,kw OR hyperemia*:ti,ab,kw OR hyperglycem*:ti,ab,kw OR 'hyper glycem*':ti,ab,kw OR hyperinsulin*:ti,ab,kw OR 'hyper insulin*':ti,ab,kw OR hyperlipidemia:ti,ab,kw OR hyperlipidemias:ti,ab,kw OR hyperlipemia:ti,ab,kw OR hyperlipemias:ti,ab,kw OR hypernephroma*:ti,ab,kw OR hypernephroid:ti,ab,kw OR 'hyper tension*':ti,ab,kw OR hypertension*:ti,ab,kw OR hypertensive*:ti,ab,kw OR 'hyper triglyceridemia':ti,ab,kw OR hypertriglyceridemia:ti,ab,kw OR 'hyper triglyceridemias':ti,ab,kw OR hypertriglyceridemias:ti,ab,kw OR hypertrophic:ti,ab,kw OR hypertrophy:ti,ab,kw OR 'impaired glucose tolerance':ti,ab,kw OR 'increased blood pressure':ti,ab,kw OR 'insulin coma*':ti,ab,kw OR 'insulin resistance':ti,ab,kw OR 'insulin resistances':ti,ab,kw OR 'insulin sensitivit*':ti,ab,kw OR 'insulin shock*':ti,ab,kw OR interatrial:ti,ab,kw OR interventricular:ti,ab,kw OR intracardiac:ti,ab,kw OR irrt:ti,ab,kw OR IRRTs:ti,ab,kw OR kidney*:ti,ab,kw OR lipedema*:ti,ab,kw OR lipemia*:ti,ab,kw OR lipidaemia*:ti,ab,kw OR lipidema*:ti,ab,kw OR lipidemia*:ti,ab,kw OR lipoedema*:ti,ab,kw OR 'lipo proteinemia*':ti,ab,kw OR lipoproteinemia*:ti,ab,kw OR 'metabolic':ti,ab,kw OR 'metabolism disorder':ti,ab,kw OR 'metabolism disorders':ti,ab,kw OR moyamoya:ti,ab,kw OR myocard*:ti,ab,kw OR myopericard*:ti,ab,kw OR nephrect*:ti,ab,kw OR nephrit*:ti,ab,kw OR nephro*:ti,ab,kw OR nicoderm:ti,ab,kw OR nicotine:ti,ab,kw OR nicotinic:ti,ab,kw OR obes*:ti,ab,kw OR overweight*:ti,ab,kw OR 'over weight*':ti,ab,kw OR pacemaker:ti,ab,kw OR pacemakers:ti,ab,kw OR parasystole:ti,ab,kw OR parasystolic:ti,ab,kw OR periarterial:ti,ab,kw OR periarteritis:ti,ab,kw OR pericard*:ti,ab,kw OR perineph*:ti,ab,kw OR perirenal*:ti,ab,kw OR pirrt:ti,ab,kw OR pirrts:ti,ab,kw OR polyarteritis:ti,ab,kw OR prediabet*:ti,ab,kw OR prehypertens*:ti,ab,kw OR 'prosthetic valve':ti,ab,kw OR 'prosthetic valves':ti,ab,kw OR pulmonary:ti,ab,kw OR pyeloneph*:ti,ab,kw OR 'qt syndrome':ti,ab,kw OR quadricuspid:ti,ab,kw OR renal*:ti,ab,kw OR reninoma*:ti,ab,kw OR renocardiac:ti,ab,kw OR renodialysis:ti,ab,kw OR "reno scleroses":ti,ab,kw OR renoscleroses:ti,ab,kw OR "reno sclerosis":ti,ab,kw OR renosclerosis:ti,ab,kw OR renotubular:ti,ab,kw OR 'reno tubular':ti,ab,kw OR renovascular:ti,ab,kw OR 'reno vascular':ti,ab,kw OR rrt:ti,ab,kw OR rrts:ti,ab,kw OR sinoatrial*:ti,ab,kw OR smoke:ti,ab,kw OR smoker:ti,ab,kw OR smokers:ti,ab,kw OR smokes:ti,ab,kw OR smoking:ti,ab,kw OR snuff:ti,ab,kw OR snuffing:ti,ab,kw OR snuffs:ti,ab,kw OR stroke:ti,ab,kw OR strokes:ti,ab,kw OR subaort*:ti,ab,kw OR supraventric*:ti,ab,kw OR 'syndrome x':ti,ab,kw OR systol*:ti,ab,kw OR tachycardia*:ti,ab,kw OR tachycardic:ti,ab,kw OR thromboembolism*:ti,ab,kw OR tobacco*:ti,ab,kw OR triatrium:ti,ab,kw OR tricuspid:ti,ab,kw OR thrombosis:ti,ab,kw OR ultrafiltration*:ti,ab,kw OR univentricular:ti,ab,kw OR uremia*:ti,ab,kw OR uremic:ti,ab,kw OR urinoma*:ti,ab,kw OR vape:ti,ab,kw OR vaper:ti,ab,kw OR vapers:ti,ab,kw OR vapes:ti,ab,kw OR vaping:ti,ab,kw OR 'vascular disease':ti,ab,kw OR 'vascular diseases':ti,ab,kw OR 'vascular disorder':ti,ab,kw OR 'vascular disorders':ti,ab,kw OR 'vascular necroses':ti,ab,kw OR 'vascular necrosis':ti,ab,kw OR 'vascular neoplasm':ti,ab,kw OR 'vascular neoplasms':ti,ab,kw OR vasculitis:ti,ab,kw OR vasculopath*:ti,ab,kw OR ventricular:ti,ab,kw)

<u>CINAHL</u>

1,129 results July 19, 2021

((MH "Emigration and Immigration") OR (MH "Immigrants+") OR (MH "Transients and Migrants") OR "African born" OR alien OR aliens OR "asylum seek*" OR "birth place" OR birthplace OR "border cross*" OR "cross border*" OR diaspora* OR emigrant OR emigrants OR emigrat* OR "foreign-born*" OR "foreign national*" OR "foreign worker*" OR foreigner* OR illegals OR immigrant OR immigrants OR immigrat* OR migrant* OR migrat* OR nomad OR nomads OR refugee OR refugees OR squatter OR squatters OR undocumented)

AND

((MH "Africa+") OR Africa* OR Algeria* OR Angola* OR Basutoland* OR Bechuanaland* OR Benin* OR black OR blacks OR Botswana* OR "Burkina Faso*" OR "Burkina Fasso*" OR Burundi* OR "Cabo Verde*" OR Cameroon* OR Cameroun* OR "Cape Verde*" OR Centrafrica* OR Centrafriqu* OR Chad* OR "Comoro Island*" OR Comoros* OR Congo* OR "Cote D'Ivoire*" OR "Cote Dilvoire*" OR Dahome* OR Djibouti* OR Egypt* OR Eritrea* OR Ethiopia* OR Eswatini* OR Gabon* OR Gaboon* OR Gambia* OR Ghana* OR "Gold Coast*" OR Guinea* OR Ifni* OR Iibia* OR "Issa land" OR "Ivory Coast*" OR Kalahari* OR Katanga* OR Kenya* OR Lesothan* OR Lesotho* OR Liberia* OR Libya* OR Madagasc* OR Maghreb* OR Maghrib* OR "Malagasy Republic" OR Malawi* OR Mali OR Malian* OR Mauretania* OR Mauritania* OR Mayott* OR Mocambiqu* OR Morocc* OR Mozambiqu* OR Namibia* OR negro OR negroes OR negros OR "NHB" OR "NHBs" OR Niger* OR Nigeria* OR Nyasaland* OR "of color" OR "of colour" OR Rhodesia* OR "Rio Muni*" OR Ruanda* OR Rwanda* OR Sahara* OR Sahel* OR "Sao Tome and Principe*" OR Senegal* OR "Sierra Leone*" OR Somali* OR Sudan* OR Swaziland* OR Tanganyika* OR Tanzania* OR Tchad* OR Togo* OR Tunesia* OR Tunisia* OR "Ubangi Shari*" OR Uganda* OR "United Arab Republic*" OR "Upper Volta*" OR Urundi* OR Zaire* OR Zambia* OR Zanzibar* OR Zimbabwe*) AND

((MH "Cardiometabolic Risk Factors") OR (MH "Cardiovascular Abnormalities+") OR (MH "Cardiovascular Diseases+") OR (MH "Cardiovascular Risk Factors+") OR (MH "Diabetes Mellitus+") OR (MH "Glucose Metabolism Disorders+") OR (MH "Glycogen Storage Disease") OR (MH "Heart+") OR (MH "Hyperlipidemia+") OR (MH "Insulin Resistance+") OR (MH "Kidney+") OR (MH "Kidney, Artificial+") OR (MH "Kidney Diseases+") OR (MH "Kidney Neoplasms+") OR (MH "Kidney Transplantation+") OR (MH "Metabolic Syndrome X+") OR (MH "Nephrostomy, Percutaneous") OR (MH "Nicotine") OR (MH "Nicotine Replacement Therapy") OR (MH "Obesity+") OR (MH "Passive Smoking") OR (MH "Renal Replacement Therapy+") OR (MH "Smoking+") OR (MH "Smoking Cessation") OR (MH "Tobacco+") OR (MH "Tobacco Use Cessation Products+") OR "abnormal blood pressure" OR "abnormal blood pressures" OR "adipose tissue hyperplas*" OR adiposity OR aneurysm OR aneurysms OR antihypertensi* OR anuria OR aorta OR aortic OR aortitis OR aortopulmonary OR arrhythmia OR arrhythmias OR arterial OR arteriosclerosis OR arteriosus OR arteriovenous OR arteritis OR asystol* OR atherogenesis OR atheromatosis OR atheroscler* OR atria OR atrial OR atrioventricular OR atrium OR bicuspid OR "blood dialyzer" OR "blood dialyzers" OR "blood pressure anomaly" OR "blood pressure anomalies" OR BMI OR BMIs OR "body mass index*" OR "body weight" OR bradycardia* OR bradycardic OR cardiac OR cardio* OR carditis OR cigar OR cigars OR cigarette OR cigarettes OR cigarillo OR cigarillos OR coronary OR corpulence OR corpulency OR corpulent OR CRRT OR CRRTs OR CVD OR CVDs OR "decreased blood pressure" OR "Dent's disease" OR "depressed blood pressure" OR dextrocardia* OR diabet* OR diafiltration OR diafiltrations OR dialyses OR dialysis OR dialyzer OR dialyzers OR diastol* OR "disorder of carbohydrate metabolism" OR "disorders of carbohydrate metabolism" OR "disorder of glycosylation" OR "disorders of glycosylation" OR dyslipaemia* OR dyslipemia OR dyslipemias OR dyslipidaemia* OR dyslipidemia* OR dyslipoidosis OR dyslipoproteinemia* OR "dysmetabolic" OR "e-cig" OR "e-cigs" OR "ecig" OR "ecigs" OR eclampsia OR "elevated blood pressure" OR "elevated blood pressures" OR "elevated cholesterol*" OR "elevated triglyceride" OR "elevated triglycerides" OR embolism OR embolisms OR endarteritis OR endocard* OR endomyocard* OR epicard* OR "fat overload syndrome" OR "fructose intolerance" OR "glomerular necros*" OR glomerulitis OR glomeruloneph* OR glomerulopath* OR glomeruloscleroses OR

glomerulosclerosis OR glomerulus OR "glucose intolerance" OR "glucose tolerance impairment" OR "glycogen storage disease" OR "glycogen storage diseases" OR glycogenoses OR glycogenosis OR "glycosylation disorder" OR "glycosylation disorders" OR glycosuria* OR glycosuric OR haemodiafiltration OR haemodiafiltrations OR haemodialysis OR haemofiltration OR haemofiltrations OR heart OR hearts OR hematuria* OR hemodiafiltration OR hemodiafiltrations OR hemodialyser OR hemodialysers OR hemodialyses OR hemodialysis OR hemodialyzer OR hemodialyzers OR hemofiltration OR hemofiltrations OR hemopericardium OR hepatorenal OR "high blood pressure" OR "high blood pressures" OR "high cholesterol*" OR hydronephro* OR "hyper cholesteremia*" OR hypercholesteremia* OR "hyper cholesterol*" OR hypercholesterol* OR hyperemia* OR hyperglycem* OR "hyper glycem*" OR hyperinsulin* OR "hyper insulin*" OR hyperlipidemia OR hyperlipidemias OR hyperlipemia OR hyperlipemias OR hypernephroma* OR hypernephroid OR "hyper tension*" OR hypertension* OR hypertensive* OR "hyper triglyceridemia" OR hypertriglyceridemia OR "hyper triglyceridemias" OR hypertriglyceridemias OR hypertrophic OR hypertrophy OR "impaired glucose tolerance" OR "increased blood pressure" OR "insulin coma*" OR "insulin resistance" OR "insulin resistances" OR "insulin sensitivit*" OR "insulin shock*" OR interatrial OR interventricular OR intracardiac OR IRRT OR IRRTs OR kidney* OR lipedema* OR lipemia* OR lipidaemia* OR lipidema* OR lipidemia* OR lipoedema* OR "lipo proteinemia*" OR lipoproteinemia* OR "metabolic" OR "metabolism disorder" OR "metabolism disorders" OR moyamoya OR myocard* OR myopericard* OR nephrect* OR nephrit* OR nephro* OR nicoderm OR nicotine OR nicotinic OR obes* OR overweight* OR "over weight*" OR pacemaker OR pacemakers OR parasystole OR parasystolic OR periarterial OR periarteritis OR pericard* OR perineph* OR perirenal* OR PIRRT OR PIRRTs OR polyarteritis OR prediabet* OR prehypertens* OR "prosthetic valve" OR "prosthetic valves" OR pulmonary OR pyeloneph* OR "QT syndrome" OR quadricuspid OR renal* OR reninoma* OR renocardiac OR renodialysis OR "reno scleroses" OR renoscleroses OR "reno sclerosis" OR renosclerosis OR renotubular OR "reno tubular" OR renovascular OR "reno vascular" OR RRT OR RRTs OR sinoatrial* OR smoke OR smoker OR smokers OR smokes OR smoking OR snuff OR snuffing OR snuffs OR stroke OR strokes OR subaort* OR supraventric* OR "syndrome x" OR systol* OR tachycardia* OR tachycardic OR thromboembolism* OR tobacco* OR triatrium OR tricuspid OR thrombosis OR ultrafiltration* OR univentricular OR uremia* OR uremic OR urinoma* OR vape OR vaper OR vapers OR vapes OR vaping OR "vascular disease" OR "vascular diseases" OR "vascular disorder" OR "vascular disorders" OR "vascular necroses" OR "vascular necrosis" OR "vascular neoplasm" OR "vascular neoplasms" OR vasculitis OR vasculopath* OR ventricular)

Cochrane

Results July 19, 2021: 256 reviews, 28 protocols, 108 trials

([mh "Emigrants and Immigrants"] OR [mh "Emigration and Immigration"] OR [mh "Transients and Migrants"] OR "African born" OR alien OR aliens OR (asylum NEXT/1 seek*) OR "birth place" OR birthplace OR (border NEXT/1 cross*) OR (cross NEXT/1 border*) OR diaspora* OR emigrant OR emigrants OR emigrat* OR (foreign NEXT/2 born*) OR (foreign NEXT/1 national*) OR (foreign NEXT/1 worker*) OR foreigner* OR illegals OR immigrant OR immigrants OR immigrat* OR migrant* OR migrat* OR nomad OR nomads OR refugee OR refugees OR squatter OR squatters OR undocumented)

AND

([mh "Africa"] OR Africa* OR Algeria* OR Angola* OR Basutoland* OR Bechuanaland* OR Benin* OR black OR blacks OR Botswana* OR (Burkina NEXT/1 Faso*) OR (Burkina NEXT/1 Fasso*) OR Burundi* OR (Cabo NEXT/1 Verde*) OR Cameroon* OR Cameroun* OR (Cape NEXT/1 Verde*) OR Centrafrica* OR Centrafriqu* OR Chad* OR (Comoro NEXT/1 Island*) OR Comoros* OR Congo* OR (Cote NEXT/1 D'Ivoire*) OR (Cote NEXT/1 Dilvoire*) OR Dahome* OR Djibouti* OR Egypt* OR Eritrea* OR Ethiopia* OR Eswatini* OR Gabon* OR Gaboon* OR Gambia* OR Ghana* OR (Gold NEXT/1 Coast*) OR Guinea* OR Ifni* OR libia* OR "Issa land" OR (Ivory NEXT/1 Coast*) OR Kalahari* OR Katanga* OR Kenya* OR Lesothan* OR Lesotho* OR Liberia* OR Libya* OR Madagasc* OR Maghreb* OR Maghrib* OR "Malagasy Republic" OR Malawi* OR Mali OR Malian* OR Mauretania* OR Mauritania* OR Mayott* OR Mocambiqu* OR Morocc* OR Mozambiqu* OR Namibia* OR negro OR negroes OR negros OR "NHB" OR "NHBs" OR Niger* OR Nigeria* OR Nyasaland* OR "of color" OR "of colour" OR Rhodesia* OR (Rio NEXT/1 Muni*) OR Ruanda* OR Rwanda* OR Sahara* OR Sahel* OR ("Sao Tome" NEXT/2 Principe*) OR Senegal* OR (Sierra NEXT/1 Leone*) OR Somali* OR Sudan* OR Swaziland* OR Tanganyika* OR Tanzania* OR Tchad* OR Togo* OR Tunesia* OR Tunisia* OR (Ubangi NEXT/1 Shari*) OR Uganda* OR ("United Arab" NEXT/2 Republic*) OR (Upper NEXT/1 Volta*) OR Urundi* OR Zaire* OR Zambia* OR Zanzibar* OR Zimbabwe*)

AND

([mh "Cardiometabolic Risk Factors"] OR [mh "Cardiovascular Diseases"] OR [mh "Cardiovascular Infections"] OR [mh "Diabetes Mellitus"] OR [mh "Dyslipidemias"] OR [mh "Fused Kidney"] OR [mh "Glucose Metabolism Disorders"] OR [mh "Glycogen Storage Disease"] OR [mh "Heart"] OR [mh "Heart Disease Risk Factors"] OR [mh "Insulin Resistance"] OR [mh "Kidney"] OR [mh "Kidneys, Artificial"] OR [mh "Kidney Diseases"] OR [mh "Kidney Neoplasms"] OR [mh "Kidney Transplantation"] OR [mh "Metabolic Syndrome"] OR [mh "Nephrostomy, Percutaneous"] OR [mh "Nicotine"] OR [mh "Overweight"] OR [mh "Renal Replacement Therapy"] OR [mh "Smokers"] OR [mh "Smoking"] OR [mh "Smoking Cessation"] OR [mh "Smoking Cessation Agents"] OR [mh "Smoking Devices"] OR [mh "Smoking Prevention"] OR [mh "Smoking Reduction"] OR [mh "Tobacco"] OR [mh "Tobacco Use Cessation Devices"] OR "abnormal blood pressure" OR "abnormal blood pressures" OR ("adipose tissue" NEXT/1 hyperplas*) OR adiposity OR aneurysm OR aneurysms OR antihypertensi* OR anuria OR aorta OR aortic OR aortitis OR aortopulmonary OR arrhythmia OR arrhythmias OR arterial OR arteriosclerosis OR arteriosus OR arteriovenous OR arteritis OR asystol* OR atherogenesis OR atheromatosis OR atheroscler* OR atria OR atrial OR atrioventricular OR atrium OR bicuspid OR "blood dialyzer" OR "blood dialyzers" OR "blood pressure anomaly" OR "blood pressure anomalies" OR BMI OR BMIs OR ("body mass" NEXT/1 index*) OR "body weight" OR bradycardia* OR bradycardic OR cardiac OR cardio* OR carditis OR cigar OR cigars OR cigarette OR cigarettes OR cigarillo OR cigarillos OR coronary OR corpulence OR corpulency OR corpulent OR CRRT OR CRRTs OR CVD OR CVDs OR "decreased blood pressure" OR "Dent's disease" OR "depressed blood pressure" OR dextrocardia* OR diabet* OR diafiltration OR diafiltrations OR dialyses OR dialysis OR dialyzer OR dialyzers OR diastol* OR "disorder of carbohydrate metabolism" OR "disorders of carbohydrate metabolism" OR "disorder of glycosylation" OR "disorders of glycosylation" OR dyslipaemia* OR dyslipemia OR dyslipemias OR dyslipidaemia* OR dyslipidemia* OR dyslipoidosis OR dyslipoproteinemia* OR "dysmetabolic" OR "e-cig" OR "e-cigs" OR "ecig" OR "ecigs" OR eclampsia OR "elevated blood pressure" OR "elevated blood pressures" OR (elevated NEXT/1 cholesterol*) OR "elevated triglyceride" OR "elevated triglycerides" OR embolism OR embolisms OR endarteritis OR endocard* OR endomyocard* OR epicard* OR "fat overload syndrome" OR "fructose intolerance" OR (glomerular NEXT/1 necros*) OR glomerulitis OR glomeruloneph* OR glomerulopath* OR glomeruloscleroses OR glomerulosclerosis OR glomerulus OR "glucose intolerance" OR "glucose tolerance impairment" OR "glycogen storage disease" OR "glycogen storage diseases" OR glycogenoses OR glycogenosis OR "glycosylation disorder" OR "glycosylation disorders" OR glycosuria* OR glycosuric OR haemodiafiltration OR haemodiafiltrations OR haemodialysis OR haemofiltration OR haemofiltrations OR heart OR hearts OR hematuria* OR hemodiafiltration OR hemodiafiltrations OR hemodialyser OR hemodialysers OR hemodialyses OR hemodialysis OR hemodialyzer OR hemodialyzers OR hemofiltration OR hemofiltrations OR hemopericardium OR hepatorenal OR "high blood pressure" OR "high blood pressures" OR (high NEXT/1 cholesterol*) OR hydronephro* OR (hyper NEXT/1 cholesteremia*) OR hypercholesteremia* OR (hyper NEXT/1 cholesterol*) OR hypercholesterol* OR hyperemia* OR hyperglycem* OR (hyper NEXT/1 glycem*) OR hyperinsulin* OR (hyper NEXT/1 insulin*) OR hyperlipidemia OR hyperlipidemias OR hyperlipemia OR hyperlipemias OR hypernephroma* OR hypernephroid OR (hyper NEXT/1 tension*) OR hypertension* OR hypertensive*

OR "hyper triglyceridemia" OR hypertriglyceridemia OR "hyper triglyceridemias" OR hypertriglyceridemias OR hypertrophic OR hypertrophy OR "impaired glucose tolerance" OR "increased blood pressure" OR (insulin NEXT/1 coma*) OR "insulin resistance" OR "insulin resistances" OR (insulin NEXT/1 sensitivit*) OR (insulin NEXT/1 shock*) OR interatrial OR interventricular OR intracardiac OR IRRT OR IRRTs OR kidney* OR lipedema* OR lipemia* OR lipidaemia* OR lipidema* OR lipidemia* OR lipoedema* OR (lipo NEXT/1 proteinemia*) OR lipoproteinemia* OR "metabolic" OR "metabolism disorder" OR "metabolism disorders" OR moyamoya OR myocard* OR myopericard* OR nephrect* OR nephrit* OR nephro* OR nicoderm OR nicotine OR nicotinic OR obes* OR overweight* OR (over NEXT/1 weight*) OR pacemaker OR pacemakers OR parasystole OR parasystolic OR periarterial OR periarteritis OR pericard* OR perineph* OR perirenal* OR PIRRT OR PIRRTs OR polyarteritis OR prediabet* OR prehypertens* OR "prosthetic valve" OR "prosthetic valves" OR pulmonary OR pyeloneph* OR "QT syndrome" OR quadricuspid OR renal* OR reninoma* OR renocardiac OR renodialysis OR "reno scleroses" OR renoscleroses OR "reno sclerosis" OR renosclerosis OR renotubular OR "reno tubular" OR renovascular OR "reno vascular" OR RRT OR RRTs OR sinoatrial* OR smoke OR smoker OR smokers OR smokes OR smoking OR snuff OR snuffing OR snuffs OR stroke OR strokes OR subaort* OR supraventric* OR "syndrome x" OR systol* OR tachycardia* OR tachycardic OR thromboembolism* OR tobacco* OR triatrium OR tricuspid OR thrombosis OR ultrafiltration* OR univentricular OR uremia* OR uremic OR urinoma* OR vape OR vaper OR vapers OR vapes OR vaping OR "vascular disease" OR "vascular diseases" OR "vascular disorder" OR "vascular disorders" OR "vascular necroses" OR "vascular necrosis" OR "vascular neoplasm" OR "vascular neoplasms" OR vasculitis OR vasculopath* OR ventricular)
<u>Scopus</u><br>5,188 results July 19, 2021
TIT-ABS-KEY("African born" OR alien OR aliens OR "asylum seek*" OR "birth place" OR birthplace OR "border cross*" OR "cross border*" OR diaspora* OR emigrant OR emigrants OR emigrat* OR "foreign-born*" OR "foreign national*" OR "foreign worker*" OR foreigner* OR illegals OR immigrant OR immigrants OR immigrat* OR migrant* OR migrat* OR nomad OR nomads OR refugee OR refugees OR squatter OR squatters OR undocumented)<br>AND<br>TIT-ABS-KEY(Africa* OR Algeria* OR Angola* OR Basutoland* OR Bechuanaland* OR Benin* OR black OR blacks OR Botswana* OR "Burkina Faso*" OR "Burkina Fasso*" OR Burundi* OR "Cabo Verde*" OR Cameroon* OR Cameroun* OR "Cape Verde*" OR Centrafrica* OR Centrafriqu* OR Chad* OR "Comoro Island*" OR Comoros* OR Congo* OR "Cote D'Ivoire*" OR "Cote DIvoire*" OR Dahome* OR Djibouti* OR Egypt* OR Eritrea* OR Ethiopia* OR Eswatini* OR Gabon* OR Gaboon* OR Gambia* OR Ghana* OR "Gold Coast*" OR Guinea* OR Ifni* OR libia* OR "Issa land" OR "Ivory Coast*" OR Kalahari* OR Katanga* OR Kenya* OR Lesothan* OR Lesotho* OR Liberia* OR Libya* OR Madagasc* OR Maghreb* OR Maghrib* OR "Malagasy Republic" OR Malawi* OR Mali OR Malian* OR Mauretania* OR Mauritania* OR Mayott* OR Mocambiqu* OR Morocc* OR Mozambiqu* OR Namibia* OR negro OR negroes OR negros OR "NHB" OR "NHBs" OR Niger* OR Nigeria* OR Nyasaland* OR "of color" OR "of colour" OR Rhodesia* OR "Rio Muni*" OR Ruanda* OR Rwanda* OR Sahara* OR Sahel* OR "Sao Tome and Principe*" OR Senegal* OR "Sierra Leone*" OR Somali* OR Sudan* OR Swaziland* OR Tanganyika* OR Tanzania* OR Tchad* OR Togo* OR Tunesia* OR Tunisia* OR "Ubangi Shari*" OR Uganda* OR "United Arab Republic*" OR "Upper Volta*" OR Urundi* OR Zaire* OR Zambia* OR Zanzibar* OR Zimbabwe*)<br>AND<br>TIT-ABS-KEY("abnormal blood pressure" OR "abnormal blood pressures" OR "adipose tissue hyperplas*" OR adiposity OR aneurysm OR aneurysms OR antihypertensi* OR anuria OR aorta OR aortic OR aortitis OR aortopulmonary OR arrhythmia OR arrhythmias OR arterial OR arteriosclerosis OR arteriosus OR arteriovenous OR arteritis OR asystol* OR atherogenesis OR atheromatosis OR atheroscler* OR atria OR atrial OR atrioventricular OR atrium OR bicuspid OR "blood dialyzer" OR "blood dialyzers" OR "blood pressure anomaly" OR "blood pressure anomalies" OR BMI OR BMIs OR "body mass index*" OR "body weight" OR bradycardia* OR bradycardic OR cardiac OR cardio* OR carditis OR cigar OR cigars OR cigarette OR cigarettes OR cigarillo OR cigarillos OR coronary OR corpulence OR corpulency OR corpulent OR CRRT OR CRRTs OR CVD OR CVDs OR "decreased blood pressure" OR "Dent's disease" OR "depressed blood pressure" OR dextrocardia* OR diabet* OR diafiltration OR diafiltrations OR dialyses OR dialysis OR dialyzer OR dialyzers OR diastol* OR "disorder of carbohydrate metabolism" OR "disorders of carbohydrate metabolism" OR "disorder of glycosylation" OR "disorders of glycosylation" OR dyslipaemia* OR dyslipemia OR dyslipemias OR dyslipidaemia* OR dyslipidemia* OR dyslipoidosis OR dyslipoproteinemia* OR "dysmetabolic" OR "e-cig" OR "e-cigs" OR "ecig" OR "ecigs" OR eclampsia OR "elevated blood pressure" OR "elevated blood pressures" OR "elevated cholesterol*" OR "elevated triglyceride" OR "elevated triglycerides" OR embolism OR embolisms OR endarteritis OR endocard* OR endomyocard* OR epicard* OR "fat overload syndrome" OR "fructose intolerance" OR "glomerular necros*" OR glomerulitis OR glomeruloneph* OR glomerulopath* OR glomeruloscleroses OR glomerulosclerosis OR glomerulus OR "glucose intolerance" OR "glucose tolerance impairment" OR "glycogen storage disease" OR "glycogen storage diseases" OR glycogenoses OR glycogenosis OR "glycosylation disorder" OR "glycosylation disorders" OR glycosuria* OR glycosuric OR haemodiafiltration OR haemodiafiltrations OR haemodialysis OR haemofiltration OR haemofiltrations OR heart OR hearts OR hematuria* OR hemodiafiltration OR hemodiafiltrations OR hemodialyser OR hemodialysers OR hemodialyses OR hemodialysis OR hemodialyzer OR hemodialyzers OR hemofiltration OR hemofiltrations OR hemopericardium OR hepatorenal OR "high blood pressure" OR "high blood pressures" OR "high cholesterol*" OR hydronephro* OR "hyper cholesteremia*" OR hypercholesteremia* OR "hyper cholesterol*" OR hypercholesterol* OR hyperemia* OR hyperglycem* OR "hyper glycem*" OR hyperinsulin* OR "hyper insulin*" OR hyperlipidemia OR hyperlipidemias OR hyperlipemia OR hyperlipemias OR hypernephroma* OR hypernephroid OR "hyper tension*" OR hypertension* OR hypertensive* OR "hyper triglyceridemia" OR hypertriglyceridemia OR "hyper triglyceridemias" OR hypertriglyceridemias OR hypertrophic OR hypertrophy OR "impaired glucose tolerance" OR "increased blood pressure" OR "insulin coma*" OR "insulin resistance" OR "insulin resistances" OR "insulin sensitivit*" OR "insulin shock*" OR interatrial OR interventricular OR intracardiac OR IRRT OR IRRTs OR kidney* OR lipedema* OR lipemia* OR lipidaemia* OR lipidema* OR lipidemia* OR lipoedema* OR "lipo proteinemia*" OR lipoproteinemia* OR "metabolic" OR "metabolism disorder" OR "metabolism disorders" OR moyamoya OR myocard* OR myopericard* OR nephrect* OR nephrit* OR nephro* OR nicoderm OR nicotine OR nicotinic OR obes* OR overweight* OR "over weight*" OR pacemaker OR pacemakers OR parasystole OR parasystolic OR periarterial OR periarteritis OR pericard* OR perineph* OR perirenal* OR PIRRT OR PIRRTs OR polyarteritis OR prediabet* OR prehypertens* OR "prosthetic valve" OR "prosthetic valves" OR pulmonary OR pyeloneph* OR "QT syndrome" OR quadricuspid OR renal* OR reninoma* OR renocardiac OR renodialysis OR "reno scleroses" OR renoscleroses OR "reno sclerosis" OR renosclerosis OR renotubular OR "reno tubular" OR renovascular OR "reno vascular" OR RRT OR RRTs OR sinoatrial* OR smoke OR smoker OR smokers OR smokes OR smoking OR snuff OR snuffing OR snuffs OR stroke OR strokes OR subaort* OR supraventric* OR "syndrome x" OR systol* OR tachycardia* OR tachycardic OR thromboembolism* OR tobacco* OR triatrium OR tricuspid OR thrombosis OR ultrafiltration* OR univentricular OR uremia* OR uremic OR urinoma* OR vape OR vaper OR vapers OR vapes OR vaping OR "vascular disease" OR "vascular diseases" OR "vascular disorder" OR "vascular disorders" OR "vascular necroses" OR "vascular necrosis" OR "vascular neoplasm" OR "vascular neoplasms" OR vasculitis OR vasculopath* OR ventricular)
<u>Web of Science</u>

3,598 results July 19, 2021
TS=("African born" OR alien OR aliens OR "asylum seek*" OR "birth place" OR birthplace OR "border cross*" OR "cross border*" OR diaspora* OR emigrant OR emigrants OR emigrat* OR "foreign-born*" OR "foreign national*" OR "foreign worker*" OR foreigner* OR illegals OR immigrant OR immigrants OR immigrat* OR migrant* OR migrat* OR nomad OR nomads OR refugee OR refugees OR squatter OR squatters OR undocumented)<br>AND<br>TS=(Africa* OR Algeria* OR Angola* OR Basutoland* OR Bechuanaland* OR Benin* OR black OR blacks OR Botswana* OR "Burkina Faso*" OR "Burkina Fasso*" OR Burundi* OR "Cabo Verde*" OR Cameroon* OR Cameroun* OR "Cape Verde*" OR Centrafrica* OR Centrafriqu* OR Chad* OR "Comoro Island*" OR Comoros* OR Congo* OR "Cote D'Ivoire*" OR "Cote DIvoire*" OR Dahome* OR Djibouti* OR Egypt* OR Eritrea* OR Ethiopia* OR Eswatini* OR Gabon* OR Gaboon* OR Gambia* OR Ghana* OR "Gold Coast*" OR Guinea* OR Ifni* OR libia* OR "Issa land" OR "Ivory Coast*" OR Kalahari* OR Katanga* OR Kenya* OR Lesothan* OR Lesotho* OR Liberia* OR Libya* OR Madagasc* OR Maghreb* OR Maghrib* OR "Malagasy Republic" OR Malawi* OR Mali OR Malian* OR Mauretania* OR Mauritania* OR Mayott* OR Mocambiqu* OR Morocc* OR Mozambiqu* OR Namibia* OR negro OR negroes OR negros OR "NHB" OR "NHBs" OR Niger* OR Nigeria* OR Nyasaland* OR "of color" OR "of colour" OR Rhodesia* OR "Rio Muni*" OR Ruanda* OR Rwanda* OR Sahara* OR Sahel* OR "Sao Tome and Principe*" OR Senegal* OR "Sierra Leone*" OR Somali* OR Sudan* OR Swaziland* OR Tanganyika* OR Tanzania* OR Tchad* OR Togo* OR Tunesia* OR Tunisia* OR "Ubangi Shari*" OR Uganda* OR "United Arab Republic*" OR "Upper Volta*" OR Urundi* OR Zaire* OR Zambia* OR Zanzibar* OR Zimbabwe*)<br>AND<br>TS=("abnormal blood pressure" OR "abnormal blood pressures" OR "adipose tissue hyperplas*" OR adiposity OR aneurysm OR aneurysms OR antihypertensi* OR anuria OR aorta OR aortic OR aortitis OR aortopulmonary OR arrhythmia OR arrhythmias OR arterial OR arteriosclerosis OR arteriosus OR arteriovenous OR arteritis OR asystol* OR atherogenesis OR atheromatosis OR atheroscler* OR atria OR atrial OR atrioventricular OR atrium OR bicuspid OR "blood dialyzer" OR "blood dialyzers" OR "blood pressure anomaly" OR "blood pressure anomalies" OR BMI OR BMIs OR "body mass index*" OR "body weight" OR bradycardia* OR bradycardic OR cardiac OR cardio* OR carditis OR cigar OR cigars OR cigarette OR cigarettes OR cigarillo OR cigarillos OR coronary OR corpulence OR corpulency OR corpulent OR CRRT OR CRRTs OR CVD OR CVDs OR "decreased blood pressure" OR "Dent's disease" OR "depressed blood pressure" OR dextrocardia* OR diabet* OR diafiltration OR diafiltrations OR dialyses OR dialysis OR dialyzer OR dialyzers OR diastol* OR "disorder of carbohydrate metabolism" OR "disorders of carbohydrate metabolism" OR "disorder of glycosylation" OR "disorders of glycosylation" OR dyslipaemia* OR dyslipemia OR dyslipemias OR dyslipidaemia* OR dyslipidemia* OR dyslipoidosis OR dyslipoproteinemia* OR "dysmetabolic" OR "e-cig" OR "e-cigs" OR "ecig" OR "ecigs" OR eclampsia OR "elevated blood pressure" OR "elevated blood pressures" OR "elevated cholesterol*" OR "elevated triglyceride" OR "elevated triglycerides" OR embolism OR embolisms OR endarteritis OR endocard* OR endomyocard* OR epicard* OR "fat overload syndrome" OR "fructose intolerance" OR "glomerular necros*" OR glomerulitis OR glomeruloneph* OR glomerulopath* OR glomeruloscleroses OR glomerulosclerosis OR glomerulus OR "glucose intolerance" OR "glucose tolerance impairment" OR "glycogen storage disease" OR "glycogen storage diseases" OR glycogenoses OR glycogenosis OR "glycosylation disorder" OR "glycosylation disorders" OR glycosuria* OR glycosuric OR haemodiafiltration OR haemodiafiltrations OR haemodialysis OR haemofiltration OR haemofiltrations OR heart OR hearts OR hematuria* OR hemodiafiltration OR hemodiafiltrations OR hemodialyser OR hemodialysers OR hemodialyses OR hemodialysis OR hemodialyzer OR hemodialyzers OR hemofiltration OR hemofiltrations OR hemopericardium OR hepatorenal OR "high blood pressure" OR "high blood pressures" OR "high cholesterol*" OR hydronephro* OR "hyper cholesteremia*" OR hypercholesteremia* OR "hyper cholesterol*" OR hypercholesterol* OR hyperemia* OR hyperglycem* OR "hyper glycem*" OR hyperinsulin* OR "hyper insulin*" OR hyperlipidemia OR hyperlipidemias OR hyperlipemia OR hyperlipemias OR hypernephroma* OR hypernephroid OR "hyper tension*" OR hypertension* OR hypertensive* OR "hyper triglyceridemia" OR hypertriglyceridemia OR "hyper triglyceridemias" OR hypertriglyceridemias OR hypertrophic OR hypertrophy OR "impaired glucose tolerance" OR "increased blood pressure" OR "insulin coma*" OR "insulin resistance" OR "insulin resistances" OR "insulin sensitivit*" OR "insulin shock*" OR interatrial OR interventricular OR intracardiac OR IRRT OR IRRTs OR kidney* OR lipedema* OR lipemia* OR lipidaemia* OR lipidema* OR lipidemia* OR lipoedema* OR "lipo proteinemia*" OR lipoproteinemia* OR "metabolic" OR "metabolism disorder" OR "metabolism disorders" OR moyamoya OR myocard* OR myopericard* OR nephrect* OR nephrit* OR nephro* OR nicoderm OR nicotine OR nicotinic OR obes* OR overweight* OR "over weight*" OR pacemaker OR pacemakers OR parasystole OR parasystolic OR periarterial OR periarteritis OR pericard* OR perineph* OR perirenal* OR PIRRT OR PIRRTs OR polyarteritis OR prediabet* OR prehypertens* OR "prosthetic valve" OR "prosthetic valves" OR pulmonary OR pyeloneph* OR "QT syndrome" OR quadricuspid OR renal* OR reninoma* OR renocardiac OR renodialysis OR "reno scleroses" OR renoscleroses OR "reno sclerosis" OR renosclerosis OR renotubular OR "reno tubular" OR renovascular OR "reno vascular" OR RRT OR RRTs OR sinoatrial* OR smoke OR smoker OR smokers OR smokes OR smoking OR snuff OR snuffing OR snuffs OR stroke OR strokes OR subaort* OR supraventric* OR "syndrome x" OR systol* OR tachycardia* OR tachycardic OR thromboembolism* OR tobacco* OR triatrium OR tricuspid OR thrombosis OR ultrafiltration* OR univentricular OR uremia* OR uremic OR urinoma* OR vape OR vaper OR vapers OR vapes OR vaping OR "vascular disease" OR "vascular diseases" OR "vascular disorder" OR "vascular disorders" OR "vascular necroses" OR "vascular necrosis" OR "vascular neoplasm" OR "vascular neoplasms" OR vasculitis OR vasculopath* OR ventricular)

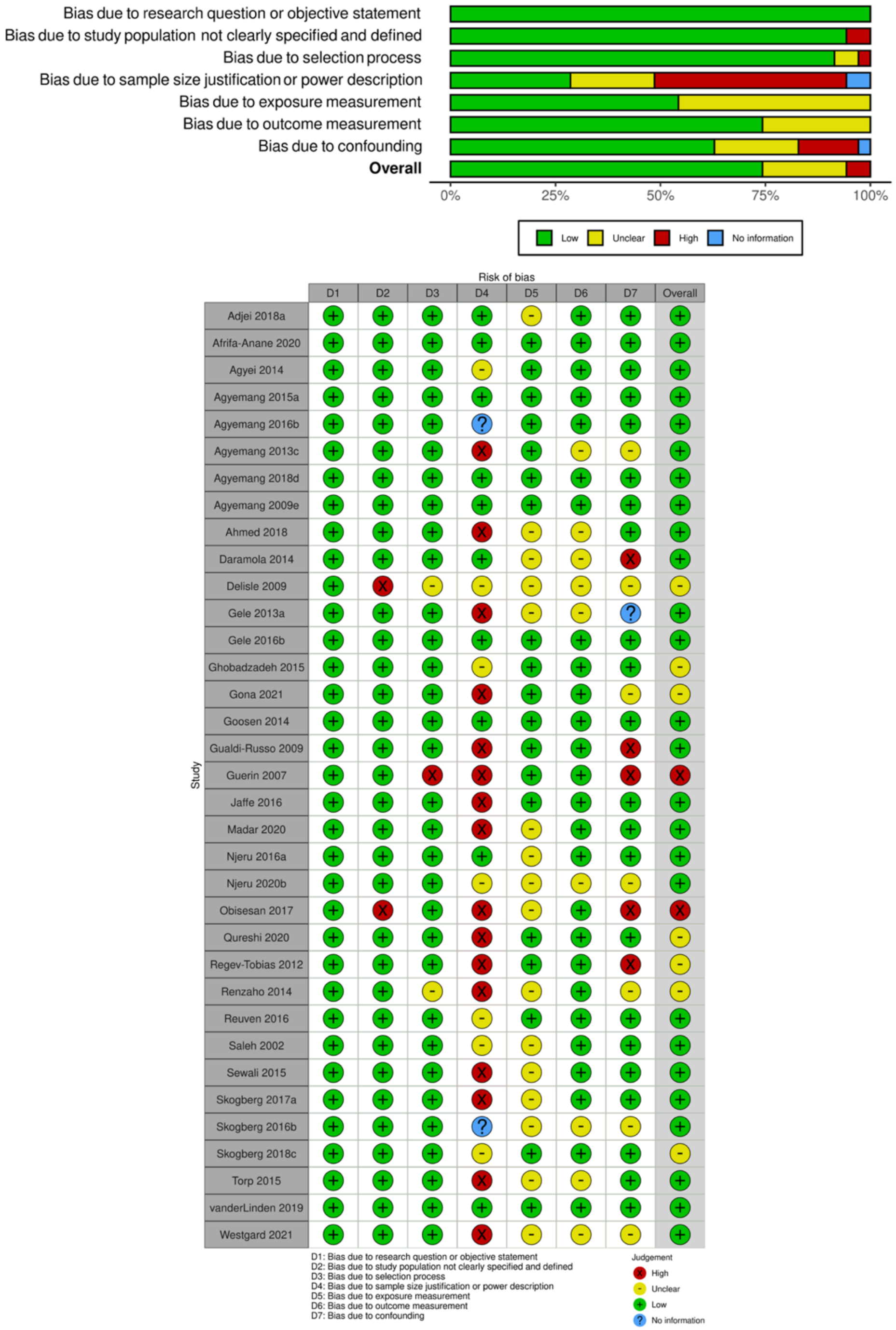

Figure S1: Risk of Bias Assessment Summary

Table S3. Prevalence and Odds Ratio of Cardiometabolic Risk Factors with African Immigrants

Author, Year	African country(ies) of origin	High Income Country	Prevalence of Cardiometabolic Risk Factor and Odds Ratio						
			Hypertension	Diabetes	Overweight/Obesity	Smoking	Dyslipidemia	Kidney Disease	CVD
**Europe**									
Adjei 2018	Ghana	Netherland<br>Germany<br>UK	Amsterdam 49.6%<br>Berlin 52.2%<br>London 51.0%	Amsterdam 13.2%<br>Berlin 14.6% London 12.8%	Amsterdam 77.7%<br>Berlin 69.0%<br>London 83.0%	Amsterdam 4.2%<br>Berlin 9.3%<br>London 0.7%	Amsterdam 11.3%<br>Berlin 12.3%<br>London 11.1%	Amsterdam 9.1%<br>Berlin 11.9%<br>London 10.3%<br>OR<br>Amsterdam: 1.37 (0.639–3.02)<br>Berlin: 1 (ref) London: 0.94	NR
Afrifa-Anane 2020	Ghana	Netherland<br>Germany<br>UK	NR	NR	Amsterdam Men 69.8% OR 0.93 (0.51–1.69)<br>Amsterdam Women 87.1% OR .91 (0.44–1.91)<br>Berlin Men 63.7% OR 1.26 (0.67–2.37)<br>Berlin Women 79.1 % OR 1.22 (0.55–2.68)<br>London Men 73.1 % OR 1.30 (0.74–2.31)<br>London Women 89.5 % OR .89 (0.48–1.69)	Amsterdam Men 18.3% OR .93 (0.44–1.89)<br>Amsterdam Women: 6.7% OR 1.09 (0.43–2.74)<br>Berlin Men 29.8% OR .87 0.45–1.68)<br>Berlin Women 9.4% OR .55 (0.17–1.78)<br>London Men 9.8% OR 1.09 (0.49–2.46)<br>London Women 2.5% OR .91 (0.27–3.04)	NR	NR	NR
Agyei 2014	Ghana	Netherlands	54.7%	NR	Overweight 41.5%,<br>Obesity: 39.2%	0.90%	NR	NR	NR
Agyemang 2015	Ghana	Netherlands	Men: 61.6%<br>PR: Men 3.57(3.17-4.02)<br>Women: 50.9%<br>Women PR: 1.90(1.72-2.09)	NR	NR	Ghanaian Men 7.8%<br>Ghanaian Female 2.4%	NR	NR	NR
Agyemang 2016	Ghana	Netherland<br>Germany<br>UK	NR	Amsterdam men 12.8% PR 3.79(2.20-6.55)<br>Amsterdam women: 9.9% PR 2.11(1.43-3.12)<br>Berlin men 15.3% PR 4.47(2.50-7.98)<br>Berlin women: 10.2% PR 2.21(1.30-3.75)<br>London men 10.4% PR 3.06(1.67-5.61)<br>London women: 8.4% PR 1.67(1.09-2.58)	Amsterdam men 18.8% PR 14.77(6.03-36.20)<br>Amsterdam women: 49.4% PR 6.00(4.58-7.84)<br>Berlin men 14.4% PR 11.41(4.48-29.04)<br>Berlin women: 39.1% PR 4.69(3.45-6.39)<br>London men 21.4% PR 15.04(5.98-37.84)<br>London women: 54.2% PR 6.63(5.04-8.72)	Amsterdam 8.1%<br>Berlin 14.8%<br>London 1.4%	NR	NR	NR
Agyemang 2013	Ghana	Netherlands	Total: 54.7 %<br>Male: 53.6 %<br>Women: 55.7%<br>Unadjusted PR: 1.04 (.81-1.33)	NR	NR	Total 0.9%<br>Men .8%<br>Women 1.0%	NR	NR	NR

Author, Year	African country(ies) of origin	High Income Country	Prevalence of Cardiometabolic Risk Factor and Odds Ratio						
			Hypertension	Diabetes	Overweight/Obesity	Smoking	Dyslipidemia	Kidney Disease	CVD
			Adjusted PR: 1.11 (.87-1.43)						
Agyemang 2018	Ghana	Netherlands	Amsterdam men 52.4% PR 1.93(1.58-2.36)<br>Amsterdam women 49.9: PR 1.45(1.25-1.68)<br>Berlin men 57.1% PR 2.21(1.78-2.73)<br>Berlin women 52.2%<br>PR 1.74(1.45-2.09)<br>London men 51% PR:1.97(1.58-2.45)<br>London women 49.3% PR: 1.51(1.28-1.78)	NR	NR	NR	NR	NR	NR
Agyemang 2009	Ghana	Netherlands	NR	NR	Total Men 69.1%<br>Men> 40 yrs 84.2%,<br>Men<40 yrs 50.0%<br>Men OR : 19.06 (8.98, 40.43)<br>Women 79.5%<br>Women>40 years 94.7%<br>Women<40 65%<br>Women OR<br>11.48 (5.97, 22.07)	NR	NR	NR	NR
Ahmed 2018	Somaliland	Norway	NR	NR	Men 9.2%<br>Women 44.1%	NR	NR	NR	NR
Delisle 2009	Equatorial Guinea	Spain	NR	NR	Men 11.0%<br>Women 23.2%	Men 4.8%<br>Women 6.2%	NR	NR	NR
Gualdi-Russo 2009	Senegal	Italy	Men: 35%	NR	Men<br>Overweight 21.2% (overweight)<br>Obese 5.3%	NR	NR	NR	NR
Gele 2013	Somalia	Norway	NR	NR	Men:28%<br>Women: 66%<br>Adjusted OR 13 (2.81-13.4)	NR	NR	NR	NR
Gele 2016	Somalia	Norway	6.3%	NR	Overweight/Obese 78.4%<br>Overweight 43.2% OR 45.7 (0.39–.52)<br>Obese 35.2% OR 6.5 (0.29–0.43)	NR	NR	NR	NR
Goosen 2014	Angola; Burundi; Democratic Republic of	Netherlands	NR	Angola men 0.7%, women 1.5%, PR	NR	NR	NR	NR	NR

Author, Year	African country(ies) of origin	High Income Country	Prevalence of Cardiometabolic Risk Factor and Odds Ratio						
			Hypertension	Diabetes	Overweight/Obesity	Smoking	Dyslipidemia	Kidney Disease	CVD
	Congo; Guinea; Sierra Leone; Somalia; Sudan			women 2.86(1.64-4.54) Burundi men 1.2 %, women: 1.2 %, PR women 2.79(1.28-5.09) Democratic Republic Congo men 1.5%, women: 1.8% PR women: 2.46(1.23-4.26) Guinea men 0.8%, women 0.3%, PR women 1.25(0.03-5.86) Sierra Leone men 0.7%, women: 1.0%, PR women: 2.80(1.12-5.49) Somalia men 2.2%, women 3.4%, PR women: 3.71(2.96-4.56) Sudan men 1.6%, women: 2.8% PR women:5.19(2.68-8.78)					
Madar 2020	Somalia	Norway	9%	5% unadjusted: 10.9 (2.79, 43.3) aOR: 4.2 (0.96, 18.63)	NR	Men 19% Women 0%	NR	NR	NR
Qureshi 2020	Eritrea; Somalia	Norway	NR	NR	Eritrea 35% Somalia 48.8% Adjusted Odds Ratio Eritrea: 2.1 (1.5, 3.0) Somali: 4.1 (2.9, 6.0)	NR	NR	NR	
Torp 2015	Somalia	Sweden	NR	NR	Overweight 36.8% Obese 14% Men 35.7% Women 72.4% Women OR 6.67, (2.435-18)	NR	NR	NR	NR
Skogberg 2017	Somalia	Finland	Men: 37.2% OR 0.72 (0.49, 1.06) Women: 22.1% OR: 0.56 (0.38, 0.82)	Men 60.4% PR: 2.67 (1.95–3.65) Women 54.4% PR: 4.52 (3.30-6.20)	Overweight Somali Men: 42.6% Somali Women: 40.3% Obese Somali Men: 4.8% Somali Women: 49.6%	Men 4.1% Women 0%	Low HDL-cholesterol Men 31.7% PR Men: 3.61 (2.10–6.21) Women 55.4% 3.41 (2.55–4.55)	NR	NR

Author, Year	African country(ies) of origin	High Income Country	Prevalence of Cardiometabolic Risk Factor and Odds Ratio						
			Hypertension	Diabetes	Overweight/Obesity	Smoking	Dyslipidemia	Kidney Disease	CVD
							Elevated Triglycerides Men 12.4% .71 (0.34–1.48) Women 5.4% .48 (0.23–1.00)		
Skogberg 2016	Somalia	Finland	Men 19.9 % PR: 0.55 (0.32-0.97) Women 13.6 % PR: 0.57 (0.33-0.97)	Men 10.0% PR: 2.32 (0.90-6.03) Women 18.6%PR :6.0 (3.17-11.33)	Men: 5.0% PR .27 (0.12-0.60) Women: 54.1% PR 2 (2.04-3.38)	Men 4.0% (0.08-0.44) Women 0%	Men 77.1% PR 1.02 (0.87-1.19 Women 77.2% PR 1.11 (0.98-1.25)	NR	NR
Skogberg 2018	Somalia	Finland	NR	Men 15.5% (8.5 to 26.7) Women 16.4% (3.4 to 10.4)	NR	NR	NR	NR	NR
Van der Linden 2019	Ghana	Netherland Germany UK	NR	NR	Amsterdam 30.3 % Berlin 29.1 % London 30.9 %	NR	High Triglycerides Amsterdam: 48.1% PR .60 (.36-1.01) Berlin men: 52.0% .60(.35-1.03) London men 47.6% .62 (.31-1.22) High LDL-C Amsterdam men: 59.3% PR 2.04 (1.64-2.53) Berlin men: 59.0% PR 1.99 (1.60-2.49) London men: 59.9% PR 1.87 (1.47-2.39)	NR	NR
**North America**									
Daramola 2014	Nigeria	United States	27.7% (Stage 1 SBP 140-159) 7.7 % (Stage 2 SBP >= 160)	NR	Women: Overweight 27.5% Women Obese 63.7%	NR	NR	NR	NR
Ghobadzadeh, 2015	Ethiopia	United States	Men 33% Women 24%	Men 9%, Women 12%	NR	NR	Total cholesterol > 240 mg/dL Men 10%, Women15% Men 22%, Women 18%	NR	NR

Author, Year	African country(ies) of origin	High Income Country	Prevalence of Cardiometabolic Risk Factor and Odds Ratio						
			**Hypertension**	**Diabetes**	**Overweight/Obesity**	**Smoking**	**Dyslipidemia**	**Kidney Disease**	**CVD**
							LDL> 160 mg/dL Men 16%, Women 20%<br>HDL <40 mg/dL men 37.4%, Women 20%		
Gona 2021	Zimbabwe	United States	Overall 34.7%<br>Men 31.8%<br>Women 35.6%	Total 8.6% Men: 6.7% Women 9.1%	Overall: 74.4%<br>Men: 60%<br>Women: 79.7%	NR	Overall 19.8%<br>Men 26.1%<br>Women 17.8%	NR	Overall 5.8%<br>Men 4.8%<br>Women 6.1%
Njeru 2016	Somalia	United States	17% Unadjusted 0.90 (0.71–1.14) Adjusted: 1.14 (0.78–1.65)	12.1% Unadjusted 2.49 (1.78–3.48) Adjusted: 2.78 (1.76–4.40)	Overweight 33.2%<br>Obese 34.6% PR<br>Unadjusted: 1.26 (1.05–1.51) Adjusted: 1.17 (0.92–1.49)	NR	18.1% PR<br>Unadjusted: 0.80 (0.64–0.998)<br>Adjusted: 0.65 (0.47–0.91)	NR	NR
Njeru 2020	Somalia	United States	NR	NR	Men Overweight 27%<br>Men Obese 14%<br>Women Overweight 31%<br>Women Obese 33%	NR	NR	NR	NR
Obisesan 2017	Nigeria	United States	NR	NR	Overweight: 28.7%<br>Obese: 38.1%	NR	NR	NR	NR
Sewali 2015	Somalia; Ethiopia; Liberia; Sudan; Kenya	United States	Somali: 5.9%<br>Ethiopia: 7.9%<br>Liberia: 15.8%<br>Sudan: 3.7%<br>Kenya: 15.7%	Somali: 4.5%<br>Ethiopia: 7.1%<br>Liberia: 5.1% Sudan: 4.8%<br>Kenya: 7.8%	Ethiopia 59.9%<br>Liberia 74.1%<br>Sudan 27.4%<br>Kenya 56.9%<br>Sudan 27.4%<br>Kenya 56.9%	NR	NR	NR	NR
Westgard 2021	Somalia	United States	Male: 29.8%<br>Female: 17.76%	Male 21.08 % Female 14.56%	Overweight<br>Women 24.54%<br>Men 28.96 %<br>Obese<br>Women 56.82 %<br>Men 23.96 %	Men 14.66%<br>Women 1.98%	Men: 49.00%<br>Women: 62.54%	NR	Female 4.86%
**Middle East**									
Jaffe 2016	Ethiopia	Israel	NR	Men 9.2%<br>Women 11.6%	NR	NR	NR	NR	NR
Regev-Tobias 2012	Ethiopia	Israel	NR	NR	Overweight 42% Obese 11%	NR	NR	NR	NR
Reuven 2016	Ethiopia	Israel	Ethiopian:18.6%<br>Ethiopian men: 18.1%<br>Ethiopian women: 15.2%	Ethiopian:17.4%<br>Ethiopian men: 16.8%<br>Ethiopian women: 17.9%	Total 18.6%<br>Male 18.1%<br>Female 19.1%	Ethiopian: 7.4%<br>Ethiopian men: 13.1%<br>Ethiopian women: 2.1%	Ethiopian: 49.9%<br>Ethiopian men: 48.6%<br>Ethiopian women: 51.6%	NR	Ischemic Heart Disease 1.7%<br><br>Peripheral Vascular Disease 0.5%
**Oceania**									

Author, Year	African country(ies) of origin	High Income Country	Prevalence of Cardiometabolic Risk Factor and Odds Ratio						
			Hypertension	Diabetes	Overweight/Obesity	Smoking	Dyslipidemia	Kidney Disease	CVD
Guerin 2007	Somalia	New Zealand	NR	NR	35.7% Overweight 35.7% Obese	NR	NR	NR	NR
Renzaho 2014	Sudan	Australia	12.4%	6.4%	Overweight 30.9% Obesity 20.1%	11.5%	NR	NR	NR
Saleh 2002	Ghana	Australia	Men: 40% Women: 17.1%	NR	Overweight Men 53.3% Women 40.0% Obese Men 17.8% Women 25.7%	NR	NR	NR	NR
PR: Prevalence Ratio; CVD: Cardiovascular Disease; aOR: Adjusted Odds Ratio									

Table S4 Prevalence and Odds Ratio of Cardiometabolic Risk Factors with Host Comparator

Author, Year	High Income Country	Host Comparator	Prevalence of Cardiometabolic Risk Factor and Odds Ratio						
			Hypertension	Diabetes	Overweight/ Obesity	Smoking	Dyslipidemia	Kidney Disease	CVD
Agyemang, 2015	Netherlands	Dutch	Men: 33.7% Women: 18.9%	NR	NR	Men: 27.0% Women: 24.2%	NR	NR	NR
Daramola 2014	United States	African Americans	16.7% (Stage 1 SBP 140-159) 11.1% (Stage 2 SBP >= 160)	NR	Overweight:38.5 % Obese: 47.4 %	NR	NR	NR	NR
Gualdi-Russo 2009	Italy	Italians	Men 33.3% Women: 23.5%	NR	Roma (Italians) Men: 32.3% Women: 12.8%	NR	NR	NR	NR
Guerin 2007	New Zealand	New Zealand	NR	NR	30.1% Overweight 19.2% Obese	NR	NR	NR	NR
Jaffe, 2016	Israel	Non-Ethiopian Jews	NR	Overall:10.3% Men: 10.7% Women: 9.7%	NR	NR	NR	NR	NR
Reuven, 2016	Israel	Native Born Israelis	Total: 17.5% Men: 20.1% Women:15.2%	Total: 13.4% Male: 14.7% Female: 11.3%	Total: 17.5 % Men: 20.1% Women: 15.2%	Total: 30.1% Men: 35.8% Women: 24.8%	Native Born Israelis Total: 55.8% Male: 56.7% Female: 54.9 %	NR	Ischemic Heart Disease: 5.4% Peripheral Vascular Disease: 1.2%
Skogberg 2017 & Skogberg 2018	Finland	Finnish	NR	Men: 6.2% Women: 3.5%	NR	NR	NR	NR	NR
Skogberg 2016	Finland	Finland General Population	Men:36.0% Women:23.9%	Men:4.3% Women:3.1%	Men:18.5% Women: 20.0%	Men:21.6% Women: 17.6%	Men: 75.9% Women:69.5%	NR	NR
PR: Prevalence Ratio; CVD: Cardiovascular Disease; aOR: Adjusted Odds Ratio									
